# Validity of AI-Based Gait Analysis for Simultaneous Measurement of Bilateral Lower Limb Kinematics Using a Single Video Camera

**DOI:** 10.3390/s23249799

**Published:** 2023-12-13

**Authors:** Takumi Ino, Mina Samukawa, Tomoya Ishida, Naofumi Wada, Yuta Koshino, Satoshi Kasahara, Harukazu Tohyama

**Affiliations:** 1 Graduate School of Health Sciences, Hokkaido University, Sapporo 0600812, Japan; ino-t@hus.ac.jp; 2 Department of Physical Therapy, Faculty of Health Sciences, Hokkaido University of Science, Sapporo 0068585, Japan; 3 Faculty of Health Sciences, Hokkaido University, Sapporo 0600812, Japan; 4 Department of Information and Computer Science, Faculty of Engineering, Hokkaido University of Science, Sapporo 0068585, Japan; wada-n@hus.ac.jp

**Keywords:** human pose estimation, 2D motion analysis, walking, markerless, motion capture, Vicon, joint kinematics, both legs, lower extremity, reliability

## Abstract

Accuracy validation of gait analysis using pose estimation with artificial intelligence (AI) remains inadequate, particularly in objective assessments of absolute error and similarity of waveform patterns. This study aimed to clarify objective measures for absolute error and waveform pattern similarity in gait analysis using pose estimation AI (OpenPose). Additionally, we investigated the feasibility of simultaneous measuring both lower limbs using a single camera from one side. We compared motion analysis data from pose estimation AI using video footage that was synchronized with a three-dimensional motion analysis device. The comparisons involved mean absolute error (MAE) and the coefficient of multiple correlation (CMC) to compare the waveform pattern similarity. The MAE ranged from 2.3 to 3.1° on the camera side and from 3.1 to 4.1° on the opposite side, with slightly higher accuracy on the camera side. Moreover, the CMC ranged from 0.936 to 0.994 on the camera side and from 0.890 to 0.988 on the opposite side, indicating a “very good to excellent” waveform similarity. Gait analysis using a single camera revealed that the precision on both sides was sufficiently robust for clinical evaluation, while measurement accuracy was slightly superior on the camera side.

## 1. Introduction

Recent advances in motion analysis using video pose estimation based on deep learning approaches have attracted considerable interest [1,2,3,4,5,6]. These are commonly referred to as markerless motion capture, which enables pose estimation from standard video footage or multiple video cameras [7]. The pose estimation technology, which does not rely on reflective makers placed on the skin, could have the advantage of eliminating errors due to skin movement and marker placement. In addition, compared to traditional marker-based optical three-dimensional motion analysis or manual two-dimensional video analysis, they have great potential to solve many problems, including cost, time, efficiency, human bias, and accuracy. Appropriate use of this technology has been reported in areas such as human posture monitoring [8,9], rehabilitation [6,10], training [11,12], and injury prevention [13]. A well-known example of a pose estimation approach is Carnegie Mellon University’s OpenPose. OpenPose, an open-source video-based human pose estimation tool, has significantly expanded the possibilities of two-dimensional motion analysis [5]. It estimates probabilistic positions of various body parts in a human figure in a video, using convolutional neural networks. It also uses the Part Affinity Fields technique to determine how body parts should be connected, enabling the capture of the body as a series of interrelated poses rather than isolated points [5]. Such technological advances have raised expectations for improved pose estimation accuracy with artificial intelligence (AI). Recent studies have reported on these accuracy validations [14,15,16], with a particular focus on gait analysis, a common clinical application [17,18,19,20,21]. Zago et al. [17] examined video setups for camera distance, resolution, and walking direction in OpenPose’s estimation and reported that maximum camera distance, high resolution, and linear walking contribute to increased accuracy. Ota et al. [18] found significant strong correlations between gait analysis using OpenPose and using a marker-based motion analysis system for the knee and ankle joint peak angles in the sagittal plane but no correlation for the hip joint. On the other hand, Yamamoto et al. [21] reported good to excellent intraclass correlation coefficients (ICC) for the sagittal plane hip and knee joint angles (0.60–0.98) but poor to fair for the ankle joint angles (<0.60). From an absolute error perspective, a study comparing smartphone cameras and three-dimensional motion analysis (3D-MA) in gait analysis reported mean absolute errors (MAEs) of the ankle—7.13°, knee—5.82°, and hip—7.73° for lower limb kinematics [20]. Stenum et al. [19] reported MAEs in the sagittal plane gait analysis comparing digital video cameras with 3D-MA as 7.4° for the ankle, 5.6° for the knee, and 4.0° for the hip. These reports generally suggest acceptable correlations, reproducibility, and error ranges for sagittal plane lower limb kinematics in gait analysis. However, there remains a lack of consensus regarding the variation in accuracy at different lower limb joints. Furthermore, questions remain regarding the true accuracy of pose estimation AI due to varying video setups in these experiments. In particular, video footage generally contains lens distortion, resulting in a fundamentally different spatial coordinate system setup compared to 3D-MA. However, to the best of the authors’ knowledge, there is no existing accuracy validation of pose estimation AI motion analysis (AI-MA) with matched video setups, especially those that correct for lens distortion in video footage.

In addition, considering its use in clinical application, this study also investigated whether the measurement accuracy differs between the limb on the camera side and the limb opposite the camera when measured with a single video camera from one side. Although OpenPose can estimate the posture of the limb on the other side of the camera [5,19], it is generally thought that the accuracy is inferior for the limb opposite the camera, as it is often obscured by the closer leg. However, in clinical settings, simultaneous measurement from both sides using two cameras has been challenging. Firstly, shooting from both sides with two cameras requires ample space on both sides of the patient. Additionally, there is a frequent issue of other patients inadvertently appearing in the footage. Therefore, in most cases, especially in sagittal plane gait analysis, the side opposite the camera is typically a wall, and only one camera is often used. If accurate simultaneous evaluation of bilateral limbs with a single camera setup becomes possible, it could have significant benefits in clinical practice.

Therefore, this study aimed to validate the accuracy of gait analysis using pose estimation AI (OpenPose) with video footage that has undergone lens distortion correction using spatial calibration information similar to that of 3D-MA systems. In addition, assuming a clinical setting for sagittal plane gait analysis, this study also evaluated the feasibility of simultaneous kinematics measurement of bilateral lower limbs using a single, one-sided camera setup. The hypothesis of this study was that measurement accuracy by correcting for camera lens distortion could potentially be improved compared to previous reports of error validation in AI-MA. In addition, it was surmised that the measurement accuracy of kinematics on the left limb (opposite camera side) might be inferior to that of the right limb (camera side).

## 2. Materials and Methods

### 2.1. Participants

This study involved 21 healthy young participants, comprising 10 males and 11 females. The participants had an average age of 20.7 ± 1.0 years, height of 165.2 ± 10.6 cm, mass of 59.6 ± 12.1 kg, and body mass index of 21.6 ± 2.6 kg/m^2^. None of the participants had any medical conditions affecting physical activity or trunk/lower extremity disorders/injuries within the 12 months preceding the study. In a pilot study involving 7 participants, the effect size was calculated from the mean error and standard deviation (SD) of both AI-MA and 3D-MA, yielding a value of 0.65. Based on this effect size (dz), an alpha error level of 0.05, and a statistical power of 0.80, the sample size required to detect a significant difference using a paired *t*-test was determined to be 21 (calculated using G*Power software ver. 3.1.9.2) [22,23]. Hence, a total of 21 participants were recruited for this study. The Institutional Review Board of Hokkaido University approved this study (IRB protocol number: 19-70-1). Written informed consent was obtained from each participant prior to their involvement in the study.

### 2.2. Gait Task

The gait task was analyzed using the steady-state gait that occurs after the initial four steps. A 10 m walkway was prepared, and gait data from the middle 5 m were used. To avoid any potential decrease in gait speed, participants were instructed to continue walking beyond the 10 m without stopping. To ensure a natural gait, participants practiced 3 to 10 times to familiarize themselves with the experimental environment and walkway. The gait speed was set to each participant’s comfortable pace to measure their most natural walking state. Three successful trials were obtained for each participant. Adequate rest was provided between trials to prevent the effects of fatigue.

### 2.3. Measurement System and Equipment

**Figure 1:**
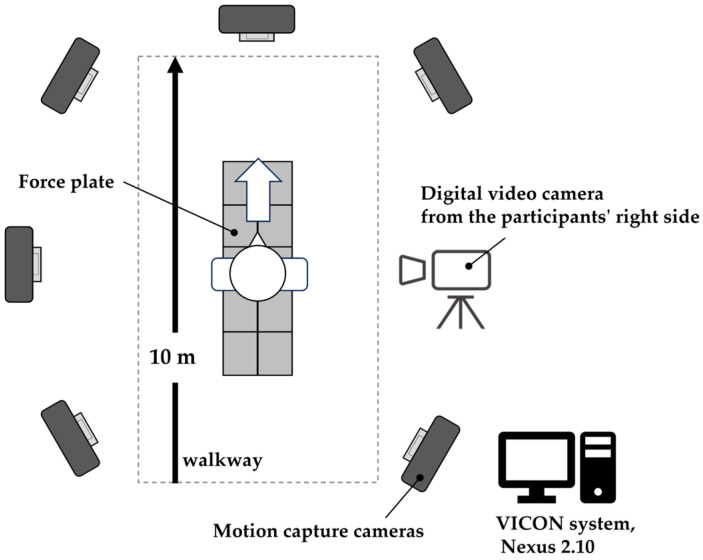
Overview of the laboratory setup.

For AI-MA, video image data were acquired using a high-speed digital video camera (Bonita Video 720C, Vicon Motion Systems Ltd., Oxford, UK) with a sampling frequency of 120 Hz. During the recording, the camera captured sagittal views from the participants’ right side to ensure accurate visualization of the motion (Figure 1).

To ensure the validity of the measurements, simultaneous 3D motion analysis (3D-MA) was conducted alongside the video measurements using the Vicon Nexus 2.10 system (Vicon Motion Systems Ltd., Oxford, UK). The Vicon motion system consisted of 14 optical cameras (Eight MX T10-S, Six Vero v2.2, Vicon Motion Systems Ltd., Oxford, UK) with a sampling frequency of 120 Hz and 10 force plates (OR6, Advanced Mechanical Technology Inc., Watertown, NY, USA) with a sampling frequency of 1200 Hz. According to the Vicon Plug-in Gait marker placement protocol, a total of 34 reflective markers were attached to specific anatomical landmarks on each participant. These markers were placed on the 7th cervical vertebra, 10th thoracic vertebra, clavicle, sternum, right scapula, and bilaterally on the front head, back head, shoulder, elbow, thumb-side wrist, pinkie side wrist, head of the second metacarpal, anterior superior iliac spine, posterior superior iliac spine, lateral thigh, lateral and medial femoral epicondyle, lateral shank, lateral and medial malleolus, the second metatarsal head, and calcaneus. Before the motion analysis trials, a static trial was conducted with each participant assuming a neutral standing position. This trial helped align the participants with the global laboratory coordinate system and establish their local joint coordinates.

### 2.4. Data Processing

**Figure 2:**
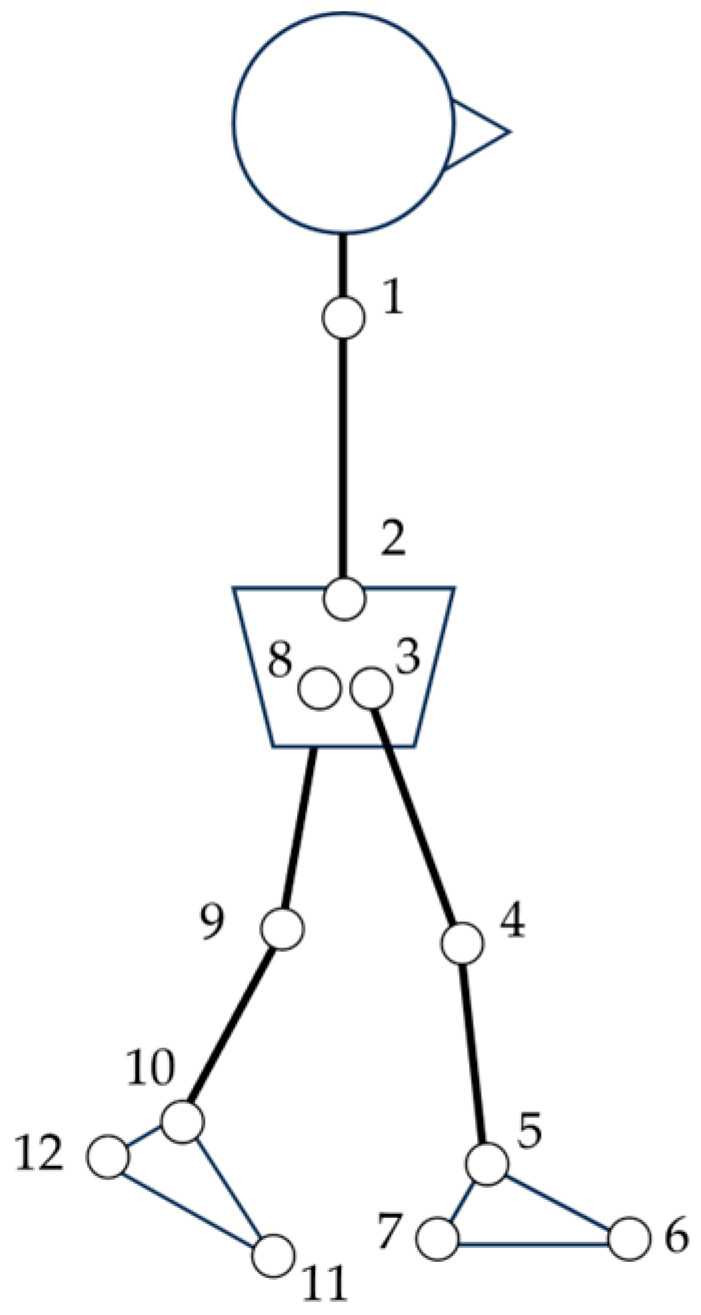
Schema of the positions of joint centers estimated by OpenPose. Feature points are as follows: 1—Neck, 2—MidHip, 3—RHip, 4—RKnee, 5—RAnkle, 6—RBigToe, 7—RHeel, 8—LHip, 9—LKnee, 10—LAnkle, 11—LBigToe, 12—LHeel. Definition of joint angles is as follows: the ankle joint angle is defined as the angle between axes 6 and 7 and 4 and 5, and between axes 11 and 12 and 9 and 10. The knee joint angle is defined as the angle between axes 3 and 4 and 4 and 5, and between axes 8 and 9 and 9 and 10. The hip joint angle is defined as the angle between axes 1 and 2 and 3 and 4, and between axes 1 and 2 and 8 and 9.

**Figure 3:**
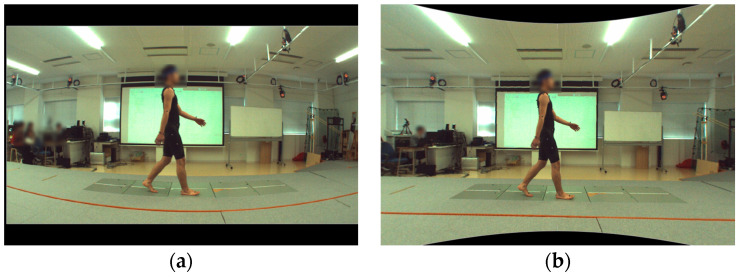
Lens distortion correction by Vicon Nexus version of 2.10. using calibration information from the Vicon system. (**a**) Pre-correction; (**b**) post-correction.

The AI-MA tool, specifically OpenPose version 1.7.0 [24], was utilized to estimate the positions of joint centers in each frame of the video (Figure 2). To eliminate the impact of lens distortion resulting from the video camera, video distortion was corrected using spatial calibration information obtained from the 3D-MA system (Figure 3). Lens distortion correction was conducted by calibrating the optical motion analysis cameras using a T-shaped wand with optical markers. A high-speed camera synchronized to the motion analysis system was calibrated simultaneously. Each calibration was used to capture 2000 and 1000 frames of the wand, respectively. Finally, using the spatial information obtained during calibration, any distortion in the video footage was corrected using the Vicon Nexus 2.10 system (Vicon Motion Systems Ltd., Oxford, UK) and then exported as a video file. This correction enabled the extraction of identification errors exclusively for AI-MA in this study. The trajectories of joint positions were subjected to filtering using a fourth-order Butterworth low-pass filter with zero lag and a cutoff frequency of 6 Hz. On the two-dimensional plane obtained, coordinates for the hip, knee, and ankle joint centers were estimated.

In the reference assessment using 3D-MA, the coordinates of the hip, knee, and ankle joint centers were determined based on the marker coordinates obtained from the markers placed on the participants’ body [25]. The trajectories of the markers were also filtered using a zero-lag, fourth-order Butterworth low-pass filter with a cutoff frequency of 6 Hz, similar to the filtering applied in the video analysis. This ensured consistency in the processing of the marker data and facilitated a reliable comparison between the video analysis and the reference 3D-MA.

### 2.5. Data Analysis and Statistics

The analysis interval was defined as a gait cycle, from the initial contact to the next initial contact. Ground contact was determined when the vertical component of the ground reaction force data exceeded 20 N. Additionally, the stance and swing phases were identified from the ground reaction force data. This same analysis interval was also applied to synchronized video analysis in AI-MA. Within this interval, the following parameters were calculated for each joint angle: (1) ankle joint, peak dorsiflexion angle, peak plantarflexion angle, and dorsiflexion–plantarflexion angular excursion; (2) knee joint, stance phase peak flexion angle, stance phase peak extension angle, stance phase flexion–extension angular excursion, swing phase peak flexion angle, swing phase peak extension angle, and swing phase flexion–extension angular excursion; (3) hip joint, peak flexion angle, peak extension angle, and flexion–extension angular excursion. To verify the reproducibility of joint kinematics across the three gait trials, the intraclass correlation coefficient (ICC) (1, 3) and its 95% confidence intervals (95%CI) were calculated for both AI-MA and 3D-MA. The ICC is interpreted as follows [26]: ICC ≥ 0.75 (excellent), 0.4 ≤ ICC < 0.75 (fair to good), and ICC < 0.4 (poor). To assess the accuracy of AI-MA compared to 3D-MA, the mean absolute error (MAE) with a 95% CI was calculated for both AI-MA and human-MA using the 3D-MA results as a reference. The interpretation of absolute error (AE) was categorized into different ranges: AE ≤ 2° (good accuracy), 2° < AE ≤ 5° (acceptable accuracy), 5° < AE ≤ 10° (tolerable accuracy), and AE > 10° (unacceptable accuracy) [27,28], based on previous studies. Additionally, Pearson’s correlation coefficient was calculated between the joint parameters obtained from AI-MA and those obtained from 3D-MA. Furthermore, the coefficient of multiple correlation (CMC) was analyzed to compare the waveform pattern similarity [29] between AI-MA and 3D-MA. CMC values were interpreted as follows: 0.65–0.75 (moderate), 0.75–0.85 (good), 0.85–0.95 (very good), and 0.95–1.00 (excellent) [30]. Paired *t*-tests were conducted to compare MAEs, CMCs, and Pearson’s correlation coefficients between AI-MA and 3D-MA. The statistical significance level was set at *p* < 0.05, and IBM SPSS Statistics version 22.0 (IBM Corporation, Armonk, NY, USA) was used for the statistical analyses.

## 3. Results

The ICCs (1,3) and their 95% CIs for each joint parameter during gait are shown in Table 1. The ICCs (1,3) for 3D-MA were 0.91 to 0.99 (*p* < 0.001), indicating “excellent” reproducibility of the gait trial. The ICCs (1,3) for AI-MA, except for angular excursion during the swing phase, ranged from 0.77 to 0.99 (*p* < 0.001), also indicating “excellent” reproducibility. The angular excursion of the swing phase alone had an ICC (1,3) of 0.73, indicating “fair to good” reproducibility.

Across all joints, the MAEs (with 95% CI) for both right (camera side) and left (opposite camera side) were between 2° and 5°, demonstrating acceptable accuracy (Table 2). The MAEs were significantly smaller on the right side (camera side) than on the left side (opposite camera side) (ankle: *p* < 0.001, knee: *p* < 0.001, hip: *p* = 0.013). In all joint angle parameters, no significant differences (fixed errors) were observed between AI-MA and 3D-MA (Table 3). The Pearson’s correlation coefficients for each joint angle parameter revealed moderate to strong correlations in all parameters, except for the swing phase flexion–extension angular excursion of the left knee (opposite camera side) (Table 4).

**Figure 4:**
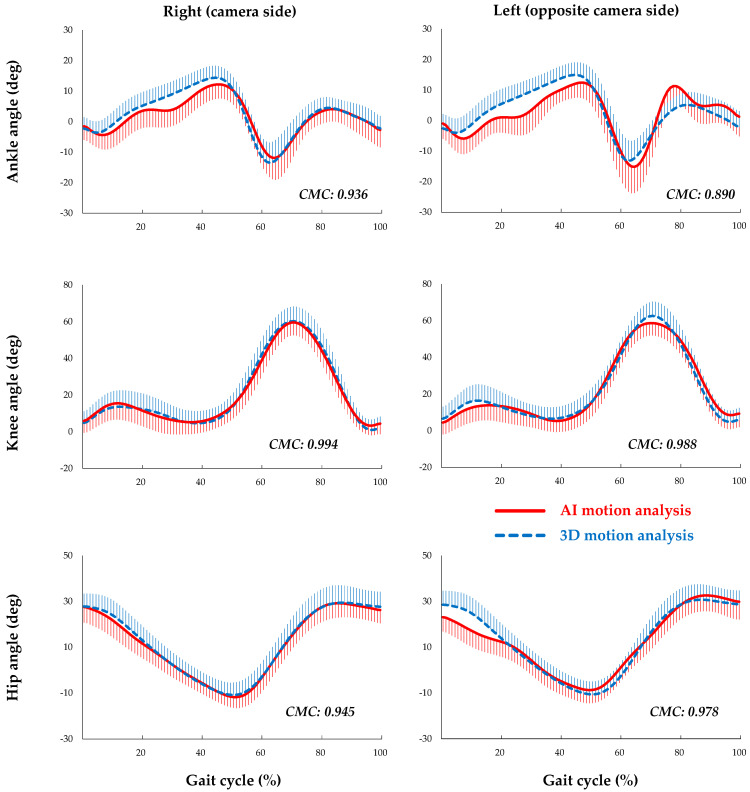
The waveform patterns in the sagittal plane of each joint during a gait cycle. Right: camera side, left: opposite camera side, red line: artificial intelligence-based (AI) motion analysis, blue-dashed line: three-dimensional (3D) motion analysis, CMC: the coefficient of multiple correlation (CMC) which indicates the waveform pattern similarity between AI and 3D motion analysis.

The waveform patterns of each joint in AI-MA and 3D-MA were closely approximated across all joints (very good to excellent) (Figure 4). The mean CMCs (SD) were as follows: for the ankle joint, the right side (camera side) was 0.936 (0.032) and the left side (opposite side of camera) was 0.890 (0.042); for the knee joint, the right side was 0.994 (0.003) and the left side was 0.988 (0.010), with significant differences between the camera side and opposite camera side (ankle: *p* < 0.001, knee: *p* = 0.002). In contrast, for the hip joint, the mean CMCs (SD) were 0.945 (0.209) on the right side and 0.978 (0.007) on the left side, with no significant difference between the camera side and opposite camera side (*p* = 0.452) (Table 5).

## 4. Discussion

This study revealed that the MAEs and 95% CI in each joint for AI-MA compared to 3D-MA were less than five degrees for both the right and left side, indicating acceptable accuracy. Additionally, the CMC for waveform pattern similarity showed high levels (very good to excellent similarity) for both the right and left side. No significant differences were observed in the peak joint angles or angular excursions between AI-MA and 3D-MA. These results suggest that AI-MA with a single video camera possesses sufficient measurement accuracy for clinical gait analysis.

In this study, the MAEs for sagittal plane lower limb kinematics were 3.1° for the ankle, 2.3° for the knee, and 2.5° for the hip. In contrast, a previous study using smartphone cameras reported the MAEs of the ankle as 7.13°, the knee as 5.82°, and the hip as 7.73° [20], and a report using digital video cameras synchronized with three-dimensional motion analysis showed 7.4° for the ankle, 5.6° for the knee, and 4.0° for the hip [19]. Therefore, the results of this study indicate a better estimation accuracy of the pose estimation AI compared to previous studies. This improvement can be attributed to the matching of the video setup (measurement frequency, resolution, and camera distance) with the reference three-dimensional motion analysis and the correction of lens distortion in the video footage. In other words, while previous studies included errors due to differences in the experimental setup and lens distortion in the video footage [19,20,21], this study was able to report more pure identification errors of the pose estimation AI. To the best of our knowledge, this study is the first to verify the error in gait analysis by AI-MA after correcting for camera lens distortion.

Another theme of this study was the measurement accuracy comparison between the right side (camera side) and left side (opposite camera side). From the perspective of the MAEs, the right side showed approximately 1° better accuracy in all joints compared to the left side. In terms of waveform pattern similarity, the right side exhibited slightly superior similarity in the ankle and knee joints. However, the differences of approximately 1° in the MAEs and less than 0.1 in the CMCs were considered clinically negligible. These findings indicate that, despite some caution being necessary, simultaneous evaluation of both limbs using a single camera from one side is feasible. These findings provide useful and reasonable evidence that could facilitate gait analysis in clinical settings and thus be clinically relevant. Furthermore, we believe that the small differences between the left and right (~1°) would have a very limited effect on gait performance indicators such as stride variability, cadence, and gait speed. Specifically, the small left–right difference is likely to have minimal effect on stride variability and cadence. Thus, it is considered that AI-based motion analysis is sufficiently useful for clinical application, and the extraction of gait indices from video footage will be an extremely valuable method in the future.

The analysis of ICCs (1,3) and their 95% confidence intervals demonstrated that the gait tasks were highly stable and reproducible (Table 1). Furthermore, the AI-MA had sufficient reproducibility. Therefore, gait analysis using AI-MA is considered practical and useful for daily functional assessment of patients.

For joint kinematics measurements during gait, the accuracy of AI-MA has been found to be comparable or superior to other existing methods [27,31,32,33,34]. In comparison with electronic goniometers and 3D-MA, Rivera et al. [32] reported that the AEs in knee flexion–extension angles during a gait cycle ranged from 2.46° to 12.49°. Similarly, Ishida et al. [31] reported the AEs of 3.3 ± 1.5° for the same parameter, and the CMCs for waveform pattern comparison of 0.992 ± 0.008. In addition, comparisons between inertial measurement unit (IMU)-based systems and 3D-MA reported differences in hip and knee joint angles during a gait cycle with a root mean squared error (RMSE) of less than 10° [27]. Another study using similar methods reported average RMSEs of 10.14°, 7.88°, and 9.75° for hip, knee, and ankle joints, respectively [34]. Shuai et al. [33] reported CMCs ranging from 0.91 to 0.98 for lower limb joint angles during gait. Compared to these other existing methods, the measurement errors of lower limb kinematics for gait analysis by AI-MA were considered equivalent or superior. 

**Figure 5:**
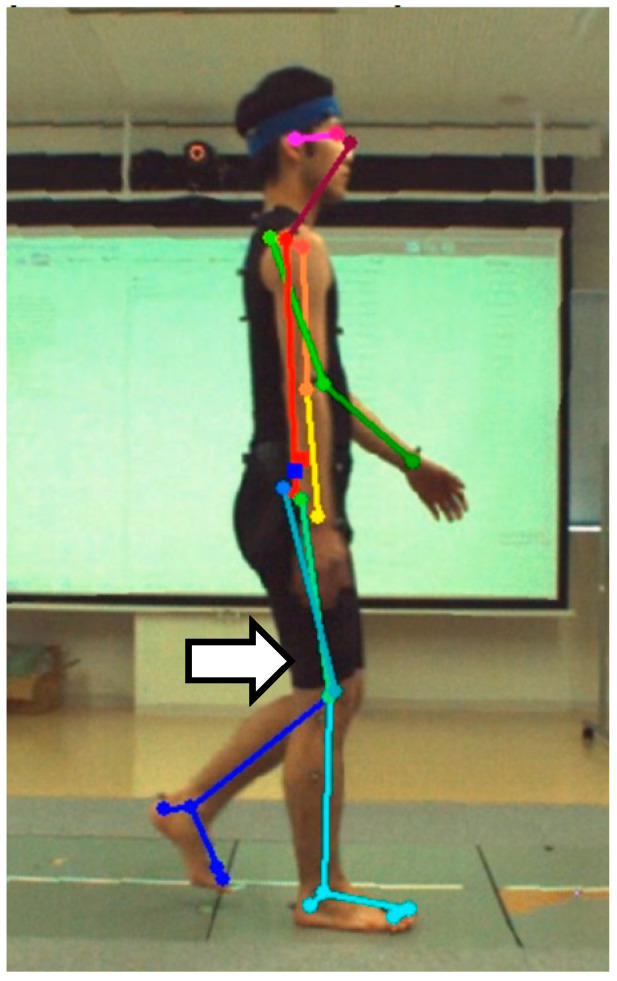
The left knee joint (blue line) is partly hidden behind the right lower limb (light blue line) during the initial swing phase.

In this study, a significant correlation was not observed only in the knee flexion–extension angular excursion of the swing phase for the left side (opposite camera side) between AI-MA and 3D-MA. One possible explanation is due to the pose estimation error. The left knee joint (opposite camera side) is partly hidden behind the right lower limb (camera side) during the initial swing phase when the peak knee flexion angle is observed (Figure 5). OpenPose, using convolutional neural networks and Part Affinity Fields technology, was considered capable of probabilistically estimating the position of body parts in a video, even when they are partially obscured [5]. However, the accuracy was found to be inferior due to deviations from actual measurements. Another reason could be the increased effect of kinematic crosstalk [35], such as tibial rotation and knee varus/valgus motion, with increasing knee flexion angle, and the possibility that the knee flexion–extension direction was not aligned with the sagittal plane due to hip internal/external rotation motions. These factors also have contributed to the small correlation coefficients which were significant not only on the left side but also on the right side (camera side).

The advantages of AI in this context are indicated as follows:

- It eliminates human work in extracting lower limb kinematics from two-dimensional videos.
- It avoids bias and human error in joint position identification.
- Compared to 3D-MA, it offers a vastly broader adaptability in terms of measurement space and environment.
- It is significantly more cost-effective than 3D-MA.

In addition, AI-MA does not require the placement of skin markers like traditional 3D-MA, significantly reducing patient burden. It is also less affected by clothing and eliminates errors associated with reapplying skin markers over multi-day assessments. We believe that AI-MA will be especially useful in clinical settings.

As for future research directions, the development of AI specializing in biomechanics is expected to improve both the accuracy and utility of AI-MA. OpenPose was not developed solely for biomechanics, and thus, it has certain limitations. Three-dimensional motion analysis using AI should be considered. Several attempts have already been made [1,15], and it is expected that skin markers will become unnecessary in the near future. However, markerless three-dimensional motion analysis has not been widely used yet. Third, the construction of big data concerning injuries as well as diseases should be considered crucial. AI-MA is a simplified motion analysis system that was developed as such due to time and cost constraints. The collection of disease-specific data using AI-MA could lead to the development of better predictive systems as well as a better understanding of the mechanisms of injuries and diseases, something invaluable for the purposes of health promotion and preventive medicine. 

Previous studies about screening tests for locomotive syndrome and its severity suggest that the evaluation of lower limb kinematics through gait analysis may be useful [36,37,38]. AI-MA analyzes human movement from video footage, making it highly compatible with various telehealth and medical services conducted through video and audio [39]. Therefore, it is considered viable for musculoskeletal health screening and gait ability assessments. The findings from this study may lay the foundation for this future development.

The limitations of this study should be addressed. First, there is a certain probability of misidentification in joint position calculation by AI pose estimation, which could be influenced by the content of machine learning. As a countermeasure, it is essential to always review the videos after AI-MA. Second, there are inherent errors in 3D-MA (Vicon) as well. Third, as a two-dimensional motion analysis, it is important to note that all joint angles are projected angles onto a single plane. Attention is needed as it does not separate errors due to three-dimensional body orientations and long-axis rotations of the limbs. In addition, it has been noted that camera distance and video resolution also affect measurement accuracy [17]. Fourth, another limitation is the lack of direct comparison with other gait analysis systems, such as IMU-based systems. Finally, this study focused on healthy participants and did not validate measurement results in patients with abnormal gait patterns. Additional validation is needed for abnormal gait patterns and patients with deformities in future studies. Despite these limitations, AI-MA can be extremely useful in a clinical context if used correctly, given its simplicity, versatility, and cost-effectiveness.

## 5. Conclusions

This study investigated the capability of pose estimation AI (OpenPose) to assess bilateral lower limb kinematics during gait analysis with a single, one-sided camera by comparing absolute errors and waveform pattern similarities with the reference marker-based three-dimensional motion analysis. The results showed that in gait analysis with pose estimation AI, the estimation accuracy of the kinematics for the limb on the far side from the camera (opposite side) was slightly inferior compared to the near side (camera side). However, both sides demonstrated good to acceptable accuracy (MAEs < 5°) and very good to excellent waveform similarity (CMCs > 0.85) compared to marker-based three-dimensional motion analysis, indicating a great potential for practical applications.

## Figures and Tables

**Table 1:** The intraclass correlation coefficient [ICC (1,3)] for each parameter in three measurements of the gait task.

	Right (Camera Side)		Left (Opposite Camera Side)	
	AI-MA	3D-MA	AI-MA	3D-MA
Ankle				
Peak dorsiflexion angle	0.84 [0.66–0.93]	0.95 [0.90–0.98]	0.77 [0.53–0.90]	0.98 [0.96–0.99]
Peak plantarflexion angle	0.92 [0.84–0.97]	0.93 [0.86–0.97]	0.93 [0.86–0.97]	0.97 [0.94–0.99]
Angular excursion	0.93 [0.84–0.97]	0.93 [0.86–0.97]	0.86 [0.71–0.94]	0.96 [0.91–0.98]
Knee				
Peak flexion angle of stance phase	0.98 [0.97–0.99]	0.99 [0.97–0.99]	0.98 [0.95–0.99]	0.99 [0.98–0.99]
Peak extension angle of stance phase	0.96 [0.92–0.98]	0.99 [0.97–0.99]	0.96 [0.91–0.98]	0.98 [0.97–0.99]
Angular excursion of stance phase	0.81 [0.62–0.92]	0.91 [0.81–0.96]	0.81 [0.61–0.92]	0.97 [0.93–0.99]
Peak flexion angle of swing phase	0.99 [0.97–0.99]	0.99 [0.97–1.00]	0.96 [0.92–0.98]	0.99 [0.97–0.99]
Peak extension angle of swing phase	0.95 [0.88–0.98]	0.98 [0.95–0.99]	0.89 [0.76–0.95]	0.97 [0.93–0.99]
Angular excursion of swing phase	0.85 [0.69–0.94]	0.94 [0.87–0.97]	0.73 [0.43–0.88]	0.91 [0.82–0.96]
Hip				
Peak flexion angle	0.97 [0.93–0.99]	0.98 [0.97–0.99]	0.99 [0.97–0.99]	0.99 [0.97–1.00]
Peak extension angle	0.96 [0.92–0.98]	0.99 [0.98–0.99]	0.96 [0.91–0.98]	0.99 [0.99–1.00]
Angular excursion	0.92 [0.84–0.97]	0.94 [0.87–0.98]	0.91 [0.81–0.96]	0.97 [0.91–0.98]

AI-MA: artificial intelligence-based motion analysis, 3D-MA: three-dimensional motion analysis. ICC (1,3) [95% confidence intervals]. *p* value was ≤0.001 for all parameters.

**Table 2:** The mean absolute error in each joint using the three-dimensional motion analysis as a reference.

	Right (Camera Side)	Left (Opposite Camera Side)	*p* Value
Ankle dorsiflexion/plantarflexion	3.1 (2.7–3.5)	4.1 (3.7–4.6)	<0.001
Knee flexion/extension	2.3 (2.1–2.6)	3.1 (2.8–3.4)	<0.001
Hip flexion/extension	2.5 (1.1–3.9)	3.5 (3.2–3.9)	0.013

Mean (95% confidence intervals), degree. *p* value by paired *t*-tests (right vs. left).

**Table 3:** Joint angle parameters during a gait cycle.

	Right (Camera Side)			Left (Opposite Camera Side)		
	AI-MA	3D-MA	*p* Value	AI-MA	3D-MA	*p* Value
Ankle						
Peak dorsiflexion angle	13.1 (4.0)	14.1 (4.2)	0.395	15.8 (3.9)	15.5 (4.2)	0.829
Peak plantarflexion angle	13.4 (6.2)	13.9 (6.1)	0.801	16.7 (7.1)	13.9 (6.2)	0.162
Angular excursion	26.5 (8.2)	28.0 (6.7)	0.503	32.5 (5.9)	29.4 (5.5)	0.078
Knee						
Stance phase peak flexion angle	14.5 (8.3)	15.7 (8.6)	0.613	15.8 (8.1)	16.6 (8.7)	0.726
Stance phase peak extension angle	2.1 (5.6)	3.4 (5.9)	0.470	1.7 (5.4)	4.5 (6.1)	0.114
Stance phase angular excursion	9.6 (3.2)	9.8 (3.5)	0.563	11.8 (3.8)	10.6 (4.1)	0.310
Swing phase peak flexion angle	61.0 (6.7)	59.7 (7.6)	0.876	59.6 (6.7)	62.3 (8.1)	0.233
Swing phase peak extension angle	0.3 (5.1)	2.8 (5.6)	0.121	7.5 (6.5)	4.3 (6.1)	0.088
Swing phase angular excursion	57.7 (4.4)	55.3 (4.1)	0.065	55.4 (3.8)	56.4 (4.2)	0.397
Hip						
Peak flexion angle	30.1 (6.3)	30.6 (6.0)	0.797	33.7 (6.5)	32.1 (6.6)	0.193
Peak extension angle	12.3 (4.6)	11.4 (5.0)	0.530	9.3 (5.5)	10.8 (5.5)	0.359
Angular excursion	42.4 (4.3)	42.0 (3.8)	0.721	43.0 (4.7)	42.0 (3.4)	0.394

AI-MA: artificial intelligence-based motion analysis, 3D-MA: three-dimensional motion analysis. Mean (SD), degree. *p* value by paired *t*-tests (right vs. left).

**Table 4:** The Pearson’s correlation coefficients for each joint angle parameter.

	Right (Camera Side)		Left (Opposite Camera Side)	
	Pearson’s *r*	*p* Value	Pearson’s *r*	*p* Value
Ankle				
Peak dorsiflexion angle	0.734	<0.001	0.857	<0.001
Peak plantarflexion angle	0.877	<0.001	0.942	<0.001
Angular excursion	0.794	<0.001	0.816	<0.001
Knee				
Peak flexion angle of stance phase	0.987	<0.001	0.957	<0.001
Peak extension angle of stance phase	0.937	<0.001	0.973	<0.001
Angular excursion of stance phase	0.806	<0.001	0.625	0.001
Peak flexion angle of swing phase	0.875	0.006	0.836	<0.001
Peak extension angle of swing phase	0.944	<0.001	0.894	<0.001
Angular excursion of swing phase	0.473	0.023	0.338	0.115
Hip				
Peak flexion angle	0.913	<0.001	0.933	<0.001
Peak extension angle	0.932	<0.001	0.954	<0.001
Angular excursion	0.598	0.003	0.716	<0.001

Pearson’s *r*: correlation coefficients between artificial intelligence-based motion analysis and three-dimensional motion analysis.

**Table 5:** The coefficient of multiple correlation (CMC) for the waveform pattern similarity in each joint.

	Right (Camera Side)	Left (Opposite Camera Side)	*p* Value
Ankle dorsiflexion/plantarflexion	0.936 (0.032)	0.890 (0.042)	<0.001
Knee flexion/extension	0.994 (0.003)	0.988 (0.010)	0.002
Hip flexion/extension	0.945 (0.209)	0.978 (0.007)	0.452

Mean (SD). CMC: artificial intelligence-based motion analysis vs. three-dimensional motion analysis. *p* value by paired *t*-tests (right vs. left).

## Data Availability

Data are contained within the article.
